# Sorptive removal of phenanthrene from aqueous solutions using magnetic and non-magnetic rice husk-derived biochars

**DOI:** 10.1098/rsos.172382

**Published:** 2018-05-30

**Authors:** Wei Guo, Shujuan Wang, Yunkai Wang, Shaoyong Lu, Yue Gao

**Affiliations:** 1 School of Environmental Science and Engineering, North China Electric Power University, Beinong Road 2, Beijing 102206, People's Republic of China; 2 National Engineering Laboratory for Lake Pollution Control and Ecological Restoration, Research Centre of Lake Environment, Chinese Research Academy of Environmental Sciences, Beijing 100012, People's Republic of China; 3 Analytical, Environmental and Geo-Chemistry, Vrije Universiteit Brussel, Pleinlaan 2, Brussel 1050, Belgium

**Keywords:** biochar, magnetic modification, rice husk, phenanthrene, adsorption mechanism

## Abstract

A magnetically modified rice husk biochar (MBC) was successfully prepared by a hydrothermal method from original biochar (BC) and subsequently used to remove phenanthrene (PHE) from aqueous solutions. The porosity, specific surface area and hydrophobicity of BC were significantly improved (approx. two times) after magnetic modification. The adsorption data fitted well to pseudo-second-order kinetic and Langmuir models. Compared with BC, MBC had a faster adsorption rate and higher adsorption capacity of PHE. The adsorption equilibrium for PHE on MBC was achieved within 1.0 h. The maximum adsorption capacity of PHE on MBC was 97.6 mg g^−1^ based on the analysis of the Sips model, which was significantly higher than that of other sources of BCs. The adsorption mechanism of the two BCs was mainly attributed to the action of surface functional groups and π–π-conjugated reactions. The adsorption of PHE on MBC mainly occurred in the functional groups of C–O and Fe_3_O_4_, but that on BC was mainly in the functional groups of –OH, N–H, C=C and C–O.

## 1. Introduction

Polycyclic aromatic hydrocarbons (PAHs) are fused-ring compounds that contain two or more rings and possess carcinogenic, mutagenic and toxic effects. As PAHs are lipophilic and difficult to be degraded by microorganisms, they pose a serious threat to human health and the environment. Wastewater discharges and petroleum spills from industries are the most important sources of PAHs in an aquatic environment [1]. Based on some previous research, the waters near the industrial zone and urban areas have shown higher concentrations of PAHs and are also at a higher risk of PAH contamination [2,3]. The problem of PAH pollution in an aquatic environment is particularly serious in developing countries such as in some of the surface waters of China [4]. Phenanthrene (PHE), as a typical tricyclic aromatic hydrocarbon possessing high hydrophilicity and certain stability, is often observed with a higher concentration in natural water and wastewater [5,6]. Therefore, it is necessary to carry out the research of pollution control of PAHs represented by PHE in water.

Adsorption by biochar (BC) is a greener and more cost-effective method to remove PAHs from water or soil [7–9]. However, the adsorption capacity of these BCs was influenced by their hydrophobicity, and decreased by their pore distribution and surface chemistry [10]. Moreover, pristine BC powders are difficult to separate from aqueous solutions after the removal treatment, thereby causing a secondary pollution problem [11,12]. Previous studies have shown that a magnetic modification of BC via aqueous Fe^2+^/Fe^3+^ solution or natural haematite treatments can effectively overcome these drawbacks [13]. Thus, magnetic modification of BCs resulted in enhanced efficiencies towards the adsorption of some metals such as Cd, Cr, Pb and As [14–16] while facilitating the recovery of the solid from the contaminated water via a filtration process with a magnet [17]. Despite these novel studies, the adsorption mechanism and the behaviour of magnetic BC-adsorbed PHE in aqueous solutions under different influence factors are not fully known.

Therefore, in the present study, magnetic BC (MBC) was obtained by magnetic modification of the original rice husk BC, and PHE was taken as the target contaminant in water. The effects of BC loading, solution pH and adsorption time on the adsorption of PHE were analysed. The changes of BC characteristics before and after magnetic modification and the adsorption mechanism of BC to PHE were investigated by means of Fourier transform infrared spectroscopy (FTIR), X-ray diffraction (XRD), X-ray photoelectron spectroscopy (XPS) and scanning electron microscopy (SEM).

## 2. Material and methods

### 2.1. Chemicals and materials

PHE with purities of over 98% were purchased from Aldrich (USA). Other chemicals including HCl, CaCl_2_, NaN_3_ and high-performance liquid chromatography (HPLC)-grade methanol were purchased from Beijing Chemical Co. Ltd, China. Deionized water was produced by using a Milli-Q system (Millipore Co., USA). The rice husk was pyrolysed at 773 K for 2 h in a tube furnace (SK-2.5-13, Beijing ZhongXing WeiYe Instrument Co., China) under a nitrogen flow rate of 10 ml min^−1^. The pyrolysed products were subsequently cooled to room temperature under the same nitrogen flow, and the resultant BC was homogenized, sieved (100-mesh) and finally stored in brown glass bottles. The BC was subsequently modified using hydrothermally synthesized magnetic iron oxide particles. As previously described by Trakal *et al*. [15], 1 g of FeSO_4_·7H_2_O was dissolved in 100 ml of water in a 500 ml beaker and a solution of NaOH (1 mol l^−1^) was slowly added under mixing until the pH value reached about 12.0. During this process, a precipitate of iron hydroxides was formed. The suspension was subsequently water diluted to 200 ml and maintained at constant temperature (353 K, water bath) for 2 h. The beaker was subsequently removed from the water bath and the as-formed magnetic iron oxide particles were washed with water until the magnetic suspension reached neutral pH. The as-obtained iron oxide was Fe_3_O_4_. With the aim to produce magnetic BC, 1 g of BC powder was thoroughly mixed in a small beaker with 2 ml of the magnetic suspension at constant temperature (353 K, water bath) for 6 h. This mixture was dried completely at 333 K for 24 h. In the process of mixing, the Fe_3_O_4_ was subsided on the surface of or into the pore structure of the BC [16]. Finally, the dried material was reground to obtain small particles. Furthermore, the experiment of iron leaching from MBC within the pH range of 2–11 was carried out to check the stability of MBC [18].

### 2.2. Characterization of biochars

The as-prepared MBC and BC samples were analysed as follows: (i) the specific surface area of the BC samples was determined on a BET analyser (ASAP 2020, Micromeritics, USA); (ii) the surface morphology and structure of the BCs were examined by SEM (S250MK3, Cambridge Co., UK); (iii) the elemental analysis of the BCs was carried out on an elemental analyser (Vario EL, German Elementar Co., Germany); (iv) the functional groups on the BCs before and after metal loading were determined by FTIR (Bruker, Germany); (v) the phase composition of the BCs was analysed by XRD (PANalytical X'Pert Pro diffractometer with a X'Celerator detector); (vi) the pH_pzc_ of the BCs was measured on a ZETASIZER 3000 HSA system (Zetasizer Nano, UK) and (vii) the binding energies of BC samples on the surface and around a depth of 10 nm were determined by XPS (Omicron Nanotechnology, Ltd), and the casaxps program was used for the analysis of the spectra.

### 2.3. Batch sorption experiment

The solutions of PHE with deionized water contained 0.01 mol l^−1^ CaCl_2_ and 200 mg l^−1^ NaN_3_ (as a bio-inhibitor). Batch mode adsorption studies were conducted by the previous study [15] to investigate the influence of some parameters used herein such as the BC dosage (0.01–1.0 g l^−1^), pH (2–11), contact time (5 min–24 h) and initial concentration (5–70 mg l^−1^) of PHE. All the batch experiments were performed with 10 ml polyethylene tubes. The mixed solutions of all batch experiments were shaken at 150 r.p.m. and 25°C for 24 h in a vertical temperature oscillation incubator (ZQPL-200, Tianjin Lai Bo Terry Instrument Equipment Co., Tianjin, China). The pH of all the batch experiments was adjusted with 0.01 M NaOH and HCl solutions. After the adsorption experiments, the solids were separated by centrifugation at 4000 r.p.m. for 10 min. The supernatant was filtered with a 0.22 µm Teflon filter. About 1 ml of supernatant was analysed for PHE by HPLC (Agilent 1200) using an ultraviolet detector at 254 nm. Owing to minimal sorption by the vials and no observed biodegradation, the amount sorbed by the sorbent was calculated by the difference in the sorbate mass in the solution. Each batch experiment involved three parallel samples, and a blank experiment was conducted following the same test procedure. The removal efficiency and the adsorbed amount of PHE at equilibrium (*q*_e_ (mg g^−1^)) were calculated using the following equations, respectively [19]:

2.1 $$\text{Removal rate}=100\%\times \frac{\left(C_{0}-C_{e}\right)}{C_{0}}$$

and

2.2 $$q_{e}=(C_{0}-C_{e})\times \frac{V}{m},$$

where *C*_0_ is the initial concentration of PHE (mg l^−1^), *C*_e_ represents the equilibrium concentration of PHE (mg l^−1^), *V* is the volume of the suspension (ml) and *m* is the weight of BC and MBC (g).

## 3. Results and discussion

### 3.1. Characterization of biochar

The BET surface area (*S*_BET_), pore volume (*V*_p_), pH, pH_pzc_, elemental composition and atomic ratio of BC before and after magnetic modification are listed in table 1. After the magnetic modification, the *S*_BET_ and *V*_p_ of BC were significantly improved, having a more than double increase, which is attributed to the loading of Fe*_x_*O*_y_* in the BC, in line with the results reported by Mohan *et al*. [20]. BC and MBC were both weakly alkaline (average pH values of 9.08 and 8.55, respectively). The two kinds of BC showed low pH_pzc_ values (less than 5.0), which confirmed they all had high acidic characteristics and a strong buffer capacity under basic environments. The higher pH_pzc_ value of BC (4.32) might be ascribed to the presence of a higher amount of basic functional groups in this BC [21]. Moreover, the magnetite surface coverage is probably responsible for the lower pH_pzc_ of MBC (3.71) [22]. Iron ions were successfully adhered to BC, and the content in MBC was 8.49%. In comparison with BC, the elemental content (C, H, O, N and S) and the ratio of O/C of MBC decreased, but the ratio of H/C increased, which indicated that the degree of the polarity and carbonization of MBC dropped and the hydrophobicity of MBC rose. During the process of BC magnetic modification, BC and iron oxide were mixed under heating, and a series of reactions of dehydration, dehydrogenation and decarboxylation in the carbon chain of the BC occurred, which resulted in the decrease of the element content such as H and O and the increase of hydrophobicity of MBC [23,24].

**Table 1.** Elemental compositions, atomic ratio, surface area, micropore volume and pH of BCs before and after magnetic modification. BC, biochar; MBC, magnetic biochar; *S*_BET_, surface area; *V*_p_, micropore volume.

biochar samples	BC	MBC
*S*_BET_ (m^2^ g^−1^)	52.1	109
*V*_p_ (cm^3^ g^−1^)	0.02	0.05
pH	9.08	8.55
pH_pzc_	4.32	3.71
C%	56.3	40.7
H%	2.05	1.65
O%	35.9	21.4
N%	0.71	0.47
S%	0.16	0.12
Fe%	—	8.49
O/C	0.48	0.39
H/C	0.44	0.49

**Figure 1.**
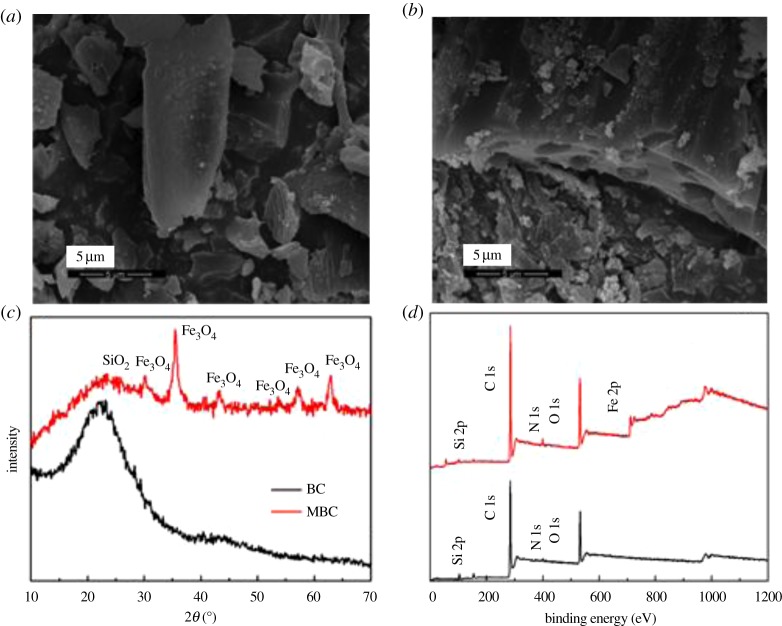
SEM images of BC (*a*) and MBC (*b*). XRD patterns (*c*) and XPS spectra (*d*) of BC and MBC.

The structure and chemical composition of BC and MBC are shown in figure 1. As shown in the SEM micrographs (figure 1*a*,*b*), the magnetic modification process resulted in a BC having a rough surface and abundance of microspheres and mineral particles, which can provide abundant adsorption points, further enhancing the adsorption capacity of the BC [16]. According to the XRD analysis, these microspheres and mineral particles in MBC might be formed by Fe_3_O_4_ (figure 1*c*). The five intense characteristic peaks detected at 2*θ* = 30.5°, 33.0°, 41.7°, 52.6° and 57.1° corresponded to the primary diffraction of the (220), (310), (400), (422) and (511) facets of a cubic spinel crystal Fe_3_O_4_ phase (JCPDS no. 19-692) [17]. Moreover, the amount of iron leaching from MBC in the solution was only 0.014 mg at pH 2, and no iron was leached from MBC within the pH range of 3–11, which confirmed that the loaded iron on the BC was really stable [18]. To further analyse the surface chemical composition of the BC samples, the XPS spectra (full scan 0–1200 eV) of BC and MBC were determined (figure 1*d*). The photoelectron lines at approximately 284, 400 and 533 eV corresponded to the C 1s, N 1s and O 1s signals of BC and MBC. Lines at binding energies of 710–721 eV corresponding to Fe 2p_3/2_ were detected for MBC and assigned to Fe^3+^ species, in line with the results reported by Trakal *et al*. [15]. This result confirmed that iron ions in the form of Fe_3_O_4_ were successfully adhered to BC via the hydrothermal synthesis method.

### 3.2 Effect of pH

**Figure 2.**
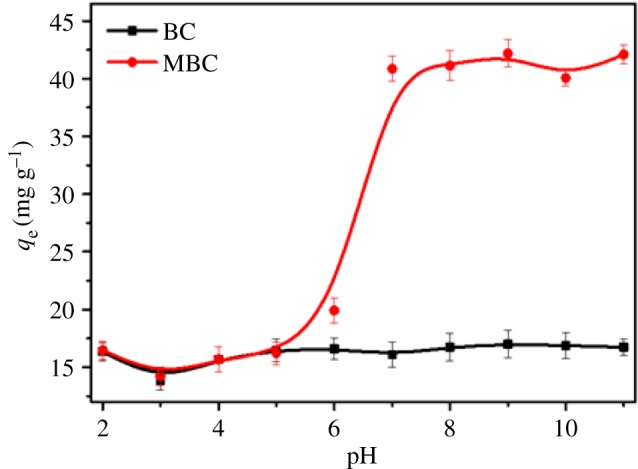
Effect of initial pH on the adsorption capacity of PHE onto BC and MBC.

**Figure 3.**
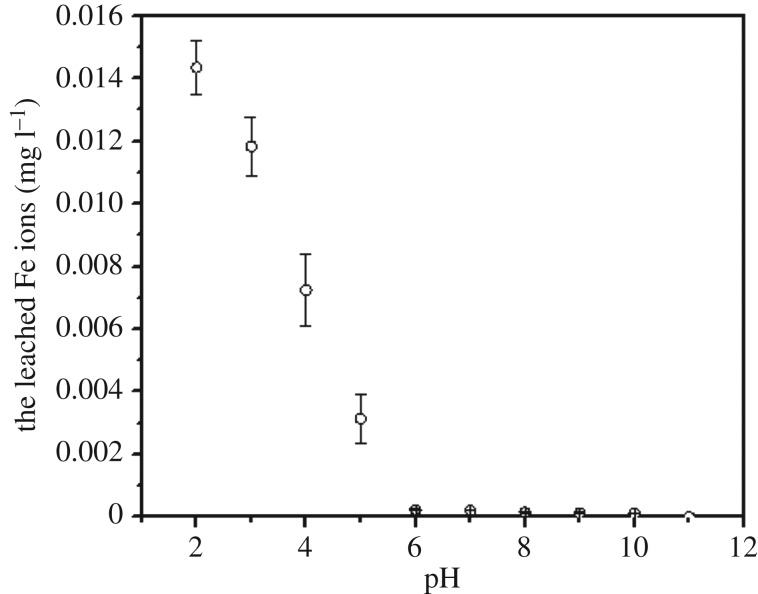
The leached characteristics of iron particles from MBC at different values of pH.

The pH of the solution affects the adsorption capacity of organic compounds due to the variation of surface charge of the adsorbent and the degree of ionization of the sorbate [25]. The effects of initial pH on the removal of PHE using BC and MBC were investigated over a pH range from 2 to 11 (figure 2). There was no significant variation for the adsorbed amounts of PHE on BC in the pH range of 2–11. The adsorption reaction of BC with these organic compounds showed its adaptive ability to a wide pH range [26,27]. PHE is a non-ionic organic compound and is not ionized in water solution. This did not show any charged area and, therefore, the adsorption of PHE was not affected by the pH change. H^+^ will only bind with BC by electrostatic attracting forces at a low pH value because both BC (pH_pzc_ = 4.32) and MBC (pH_pzc_ = 3.71) showed positive charges at this condition. Thus, pH is not a significant influencing factor for adsorption of PAHs such as PHE [28]. However, for MBC, the adsorption capacity of PHE showed an S-shaped curve with the pH change. The presence of Fe ions in MBC significantly improved the BET surface area and micropore volume of BC, which was helpful in increasing the adsorption capacity of PHE on MBC. When the solution is acidic (pH 2–6), the adsorption sites of the MBC surface are competed for by H^+^ and Fe^3+^, and at the same time, some iron ions in the surficial layer of MBC are leached due to acid dissolved action, causing the adsorptive sites of MBC to decrease (figure 3). Furthermore, these factors reduced the adsorption of MBC to the PHE. With the increase of pH value in the solution from 7 to 11, the concentration of H^+^ and the dissolved action of acid in the solution decreased gradually; thus, the surface of MBC could provide much more adsorption sites so as to improve the adsorption capacity of PHE. This showed that the molecules of PHE might have adsorbed on BC and MBC mainly via the hydrophobic partitioning interaction [29], not electrostatic action. BC and MBC both showed high removal rates for PHE at a pH of 7. Thus, this value was used for further PHE adsorption experiments on BC and MBC.

### 3.3. Adsorbent mass optimization

**Figure 4.**
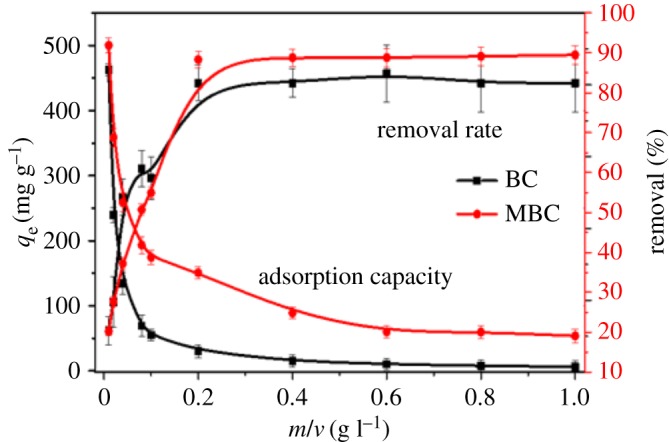
Effect of adsorbent dosage on the removal rate of PHE and adsorption capacity of BC and MBC.

The adsorbent dosage has been regarded as a crucial factor affecting the removal of contaminants [30]. The influence of the BC mass on PHE adsorption capacities is shown in figure 4. The removal rates and adsorption capacity increased and decreased, respectively, with the increase of BC dosage. The highest amount of PHE removal (greater than 85%) was attained with adsorbent masses of at least 2.0 mg. With the adsorbent dosage greater than this value, the removal rate of PHE remained almost constant. While increasing the BC loading (up to 2.0 mg) is advantageous in that it provides more active sites for adsorption, excessive dosages (greater than 2.0 mg) can lead to adsorbent aggregation issues which reduce the number of binding sites and total surface area of the adsorbent, and increase the diffusional path length [31,32]. As shown in figure 4, BC and MBC both showed high PHE removal rates at the dosage of 4.0 mg. From the economic and environmental viewpoints, 0.2 g l^−1^ was selected herein as the optimum BC loading.

### 3.4. Adsorption kinetics

Nonlinear pseudo-first-order and pseudo-second-order kinetic models [33] were used to assess the kinetics of adsorption of PHE onto BC and MBC, as shown in figure 4 and table 2. The pseudo-first-order and pseudo-second-order models can be described by the following equations [25]:

3.1 $$q_{t}=q_{e}[1-\exp(-K_{1}t)]\;(p\text{seudo-first-order})$$

and

**Figure 5.**
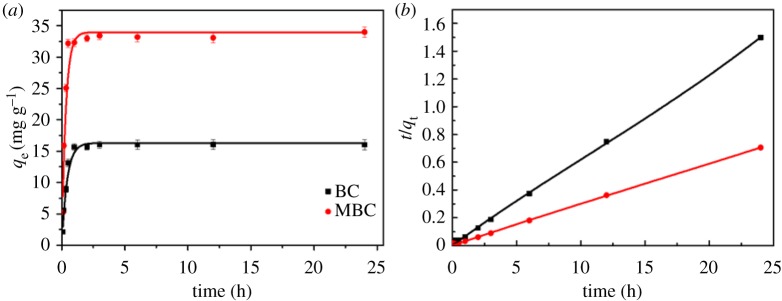
Adsorption kinetics of PHE on BC and MBC, pseudo-first-order model (*a*) and pseudo-second-order model (*b*).

3.2 $$q_{t}=q_{e}-\frac{q_{e}}{\left[K_{2}(q_{e})t+1\right]}\;(p\text{seudo-second-order}),$$

where *K*_1_ is the rate constant of the pseudo-first-order adsorption model (h^−1^), *K*_2_ is the rate constant of the pseudo-second-order adsorption model (g mg^−1^ h^−1^), and *q*_t_ and *q*_e_ (mg g^−1^) are the amounts of PHE adsorbed at a contact time *t* (h) and at equilibrium, respectively. The adsorption equilibrium of PHE on BC and MBC was obtained within 1 h, respectively (figure 5*a*). The pseudo-second-order model better described the adsorption kinetics of PHE (*R*^2 ^= 0.999) than the pseudo-first-order model (*R*^2 ^= 0.983 for BC and *R*^2 ^= 0.961 for MBC). PHE adsorption on BC was much faster than that on MBC. The rate constants of the pseudo-second-order adsorption model for PHE on BC and MBC were 0.06 g mg^−1^ h^−1^ and 0.13 g mg^−1^ h^−1^, respectively. According to the pseudo-second-order model, the reaction rate is proportional to the number of active sites on the adsorbent's surface and the rate-limiting step may be the chemical adsorption between the adsorbate and the adsorbent [26]. The equilibrium adsorption capacities (16.2 mg g^−1^ for BC and 41.0 mg g^−1^ for MBC) from the calculated value based on the pseudo-second-order adsorption model were closer to the experimental value (16.7 mg g^−1^ for BC and 42.1 mg g^−1^ for MBC), further confirming that the adsorption processes of PHE on BC and MBC are mainly physical and chemical adsorption.

**Table 2.** Kinetics and isotherm parameters for PHE adsorption on BC and MBC at 298 K. BC, biochar; MBC, magnetic biochar.

model type	BC	MBC
kinetics parameters		
pseudo-first-order		
*K*_1_ (h^−1^)	2.7	3.92
*q*_e_ (mg g^−1^)	16.1	33.6
*R*^2^	0.983	0.961
pseudo-second-order		
*K*_2_ (g mg^−1^ h^−1^)	0.06	0.13
*q*_e_ (mg g^−1^)	16.2	41.0
*R*^2^	0.999	0.999
isotherm parameters		
Langmuir		
*Q*_max_ (mg g^−1^)	55.8	89.6
*K*_L_ (l mg^−1^)	0.21	0.25
*R*^2^	0.865	0.938
Freundlich		
*K*_F_ ($\text{mg}\;{\text{g}}^{-1}{\left(\text{mg}\;{\text{l}}^{-1}\right)}^{-1/n}F$)	4.15	4.20
*n*_F_	0.83	0.68
*R*^2^	0.839	0.867
Sips		
*Q*_max_ (mg g^−1^)	65.5	97.6
*K*_s_ (l mg^−1^)	7.11	2.65
*n*_s_	2.06	2.11
*R*^2^	0.951	0.977

### 3.5. Equilibrium studies

The adsorption isotherm describes the existing relationship between the amount of adsorbate adsorbed by the adsorbent and the adsorbate concentration remaining in the solution after the system attains equilibrium at a constant temperature [25]. The adsorption isotherms of PHE on BC and MBC have been shown in figure 5. The Langmuir [34], Freundlich [35] and Sips [36] models are employed to fit the data of adsorption isotherms. The Langmuir model was often used to simulate monolayer adsorption under the assumption of adsorbent surface regularity. The Freundlich model was applied to multilayer adsorption based on the hypothesis of adsorbent surface irregularity. The Sips model is a modified type of Freundlich model; it has wider application conditions and can be better used to analyse the linear and nonlinear characteristics of the adsorption process. The relative parameters are listed in table 2. The above-mentioned models can be described by the following equations:

3.3 $$\begin{array}{rl} q_{e} & =\frac{Q_{\max}K_{L}C_{e}}{1+K_{L}C_{e}}\;(\text{Langmuir}), \end{array}$$

3.4 $$\begin{array}{rl} q_{e} & =K_{F}C_{e}^{1/n_{F}}\;(\text{Freundlich}) \end{array}$$

**Figure 6.**
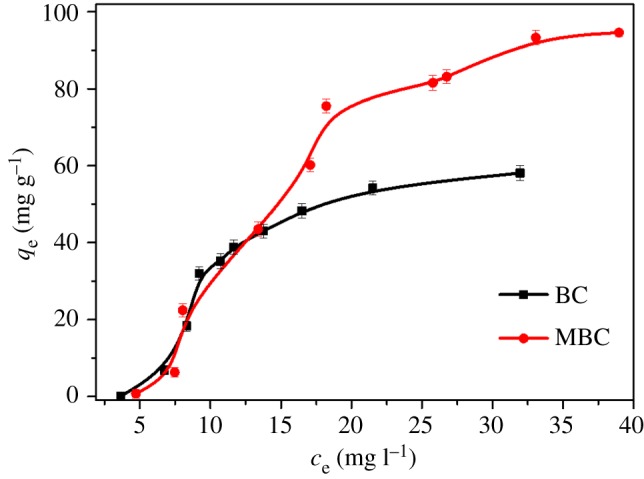
Adsorption isotherms for PHE on BC and MBC.

3.5 $$\begin{array}{rl} \;\text{and}\;q_{e} & =\frac{Q_{\max}{\left(K_{s}C_{e}\right)}^{1/n_{s}}}{1+{\left(K_{s}C_{e}\right)}^{1/n_{s}}}\;(\text{Sips}), \end{array}$$

where *C*_e_ (mg l^−1^) is the solution concentration at equilibrium, *q*_e_ (mg g^−1^) is the amount adsorbed at equilibrium, *Q*_max_ (mg g^−1^) is the maximum adsorption capacity, *K*_L_ (l mg^−1^) is the constant that is related to the free energy of adsorption, *K*_F_ is the Freundlich equilibrium constant ($\text{mg}\;{\text{g}}^{-1}{\left(\text{mg}\;{\text{l}}^{-1}\right)}^{-1/n_{F}}$), *n*_F_ is the dimensionless exponent of the Freundlich equation, which varies with the degree of heterogeneity of adsorbing sites, *K*_s_ is the Sips equilibrium constant (l mg^−1^) and *n*_s_ is the dimensionless exponent of the Sips equation. It can be seen that the trends of adsorption isotherms of PHE by BC and MBC are basically the same (figure 6). The adsorption capacity increased rapidly with the increase of the equilibrium concentration of PHE, and then slowly increased until the basic equilibrium was reached. This was because as the equilibrium concentration of PHE increased, the adsorption sites on the surface of BC decreased and then the adsorption sites gradually reached saturation [37]. In addition, the equilibrium adsorption capacity of PHE on MBC is significantly higher than that of BC, indicating that MBC had a better removal potential of PHE. In comparison with the two other models, the Sips model can better model the equilibrium data with the coefficients in the range of 0.951–0.977. According to the Sips fitting, the maximum adsorption capacity of PHE on MBC at a pH of 7.0 and a temperature of 298 K is 97.6 mg g^−1^. This value is close and even higher than the corresponding values obtained for other derived BCs reported in the literature such as 93–145 mg g^−1^ for granular activated carbon [38], 45–140 mg g^−1^ for BCs derived from orange peel and pine needle [10], 129.8 mg g^−1^ for BCs derived from pine wood chips [39] and 10.4–13.9 mg g^−1^ for graphene/BC [40]. The *n*_F_ values of the two adsorbents were both less than 1 and the MBC (0.68) < BC (0.83), indicating that MBC adsorbed PHE more easily [37]. Moreover, the value of 1/*n*_s_ is under 1, confirming the inhomogeneity of BC structure, and this trend for MBC was greater than that for BC due to the loading of iron oxides to the BC.

### 3.6. Adsorption mechanism

**Figure 7.**
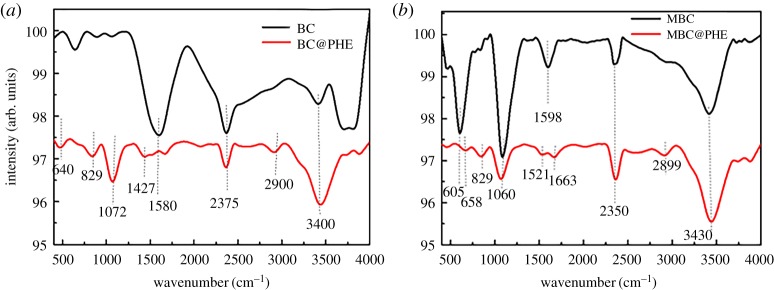
FTIR spectra of BC (*a*) and MBC (*b*) of before and after the adsorption of PHE.

To investigate the adsorption mechanism of PHE on BC and MBC, FTIR analyses were conducted before and after adsorption (figure 7). According to the results of the adsorption kinetics and adsorption isotherms, the adsorption of PHE on BC and MBC in water solution is mainly surface adsorption and π–π conjugation [39]. A band at 3400–3430 cm^−1^ was observed and ascribed to the stretching vibration of –OH of both BCs [41]. The peaks at approximately 2350–2375, 1580–1598 and 1060–1072 cm^−1^ corresponded to the –NH bond, the stretching of the C=C bond and the C–O–C vibration of BC and MBC, respectively [42]. MBC had a high stretching peak at 605 cm^−1^ and corresponded to the Fe–O bond [43]. Numerous peaks appeared or disappeared after adsorption of PHE on BC and MBC. The vibrational peaks of –OH and C–O–C at 3400 and 1072 cm^−1^ at BC increased significantly, and the peak intensities at 1580 cm^−1^ corresponding to the C=C bond decreased and shifted obviously. For MBC, the vibrational peak of 1060 cm^−1^ corresponding to C–O–C decreased significantly, and the peak intensities at 605 and 1598 cm^−1^ corresponding to the Fe–O bond and C=C bond, respectively, decreased and shifted obviously. This indicated that the adsorption of PHE on BC is related to the functional groups such as –OH, C=C and C–O–C, and that on MBC is related to functional groups such as Fe–O, C=C and C–O–C. The adsorption mechanism of PHE on BC is mainly through oxygen, hydrogen-containing functional groups and π–π interactions. In addition, a series of new peaks appeared at 2899 and 800–1000 cm^−1^ due to the C–H and other groups' vibration [44]. The PHE adsorbed on the surfaces of BC via the hydrophobic and π–π interactions, and then they would enter into the pores of the BC through a pore-filling process [45].

## 4. Conclusion

This study demonstrated that magnetically MBC could be used as a promising adsorbent of PHE. SEM, XRD and XPS results confirmed that the porosity, specific surface area and hydrophobicity of BC were significantly improved when loading iron oxides. The adsorption efficiency of MBC to PHE was significantly increased due to the change of structure and functional groups after magnetic modification. The optimum adsorption conditions were as follows: BC loading was 0.2 g l^−1^ and the pH value was above 7.0. The adsorption experimental data were well fitted by Sips isotherm and pseudo-second-order kinetic models. The maximum adsorption capacity of PHE on MBC was 97.6 mg g^−1^ based on the analysis of the Sips model. Based on the FTIR, the action of surface functional groups and π–π-conjugated reactions were suggested as the main mechanisms for the adsorption of PHE on BCs.

## Supplementary Material

BP.xlsx

## Data accessibility

All data are available in the electronic supplementary material.
